# Socioeconomic inequalities in Chile during the COVID-19 pandemic: A regional analysis of income poverty

**DOI:** 10.1371/journal.pone.0323409

**Published:** 2025-05-07

**Authors:** Iris Delgado, Sushma Dahal, Maria I. Matute, Paola A. Rubilar Ramírez, Svenn-Erik Mamelund, Gerardo Chowell

**Affiliations:** 1 Centro de Epidemiología y Política de Salud, Facultad de Medicina Clínica Alemana, Universidad del Desarrollo, Santiago, Chile; 2 School of Public Health, Georgia State University, Atlanta, Georgia, United States of America; 3 Centre for Research on Pandemics & Society (PANSOC), Oslo Metropolitan University, Oslo, Norway; 4 Department of Applied Mathematics, Kyung Hee University, Yongin, Republic of Korea; Federal University of Rio de Janeiro: Universidade Federal do Rio de Janeiro, BRAZIL

## Abstract

The COVID-19 pandemic caused an unprecedented economic crisis, intensifying poverty levels in Latin America, particularly in Chile. This study examines the short- and long-term socioeconomic impacts of COVID-19 on income poverty in Chile, focusing on regional disparities, rurality, ethnicity, educational attainment, and immigration. Using data from the Chile National Socioeconomic Characterization Survey (CASEN) for 2017, 2020, and 2022, we analyzed poverty trends across the pre-pandemic, pandemic, and post-pandemic periods. We employed spatial clustering techniques with Local Moran’s I to detect poverty hotspots and applied logistic regression models to identify key sociodemographic factors associated with these hotspots. Our results reveal stark regional disparities, with disproportionately higher poverty rates among rural populations, Indigenous communities, and individuals with lower education levels or immigrant backgrounds. The proportion of individuals in poverty hotspots rose from 6.8% in 2017 to 8.6% in 2020, before slightly declining to 7.7% in 2022. Although emergency monetary subsidies helped reduce overall poverty from 10.8% in 2020 to 6.5% in 2022, these measures were insufficient to address deep-rooted structural inequalities. Our findings underscore the urgent need for targeted, long-term policies that go beyond temporary financial assistance and tackle systemic disparities linked to rurality, ethnicity, education, and immigration. Such measures are essential for achieving sustainable poverty reduction and fostering inclusive economic growth in Chile.

## Introduction

The COVID-19 pandemic precipitated an unprecedented global economic crisis, underscoring the profound impact pandemics can have on economic stability, social structure, and inequalities. Historically, such crises have disrupted supply chains, reduced labor forces, altered consumer behaviors, intensified inequalities, and reshaped regional economies [1]. Latin America, as one of the most economically unequal regions in the world, faced a particularly severe economic contraction during the pandemic, with a sharp increase in poverty rates and worsening socioeconomic disparities [2]. Structural determinants of poverty—such as labor market informality, unequal access to education and healthcare, and geographic disparities—exacerbated the crisis, disproportionately affecting vulnerable populations [3]. Recovery efforts in 2021 and 2022 were uneven, with persistent challenges in labor market inclusion.

Chile is a compelling case due to its robust statistical system, enabling precise poverty analysis across different pandemic phases. Unlike other Latin American countries with data limitations, Chile’s high-quality datasets facilitate a granular assessment of socioeconomic trends. Furthermore, Chile’s socioeconomic landscape presents a paradox. While it boasts one of the region’s most advanced social safety nets and economic development levels, it grapples with significant structural inequalities. These disparities were exacerbated during the COVID-19 crisis, as certain groups—including Indigenous populations, informal workers, and rural communities—faced disproportionate economic hardship despite government relief measures. While Chile’s fiscal stimulus and social assistance programs successfully reduced extreme poverty post-pandemic, they were insufficient to fully address preexisting socioeconomic inequities [4,5]. However, its social insurance system differs significantly from that of its neighbors in terms of coverage, financing, and crisis response. While Chile relied on pension withdrawals and temporary cash transfers, Argentina and Brazil implemented wage subsidies and unemployment benefits, providing stronger employment protection [6]. Argentina’s Asignación Universal por Hijo (AUH) and Brazil’s Auxílio Emergencial played key roles in mitigating poverty, particularly among informal workers [7]. In contrast, Chile’s pension fund withdrawals, though providing immediate relief, may have long-term consequences for retirement security. Uruguay’s more comprehensive contributory social insurance scheme proved more resilient [8].

Before the pandemic, Chile experienced varied poverty-level trends across its regions, reflecting the complex interplay of economic activities and development policies. The northern mining regions, buoyed by high global demand for copper, saw robust economic growth and reduced poverty rates [9]. In contrast, the Araucanía southern region, with its large Indigenous population and less industrialized economy, experienced economic stagnation and increasing poverty levels. The central regions, including the capital, Santiago, faced fluctuating fortunes due to industrial growth and urban challenges [10].

This study not only examines how the socioeconomic impacts of COVID-19 on income poverty manifested in Chile but also considers how these findings extend to other Latin American countries and beyond. Chile’s experience reflects broader regional patterns, such as the vulnerability of informal workers and marginalized communities, and offers lessons for countries with less developed social insurance systems. Furthermore, the analysis is relevant to nations with more advanced social protections, underscoring the importance of addressing structural inequalities, including rurality and ethnicity, to complement social safety nets.

During the pandemic, especially in 2021, various social assistance programs were provided to low-income families, particularly monetary subsidies. As a result, poverty measured by income decreased from 10.8% in 2020 to 6.5% in 2022 [11]. However, despite the reduction in poverty, the profound economic crisis during COVID-19 highlighted and intensified the spatial heterogeneity of poverty within Chile, with regions with substantial informal economies and weaker social safety nets being the hardest hit. This pattern aligns with broader findings across Latin America, where emergency social assistance programs had varying coverage and effectiveness, particularly among informal workers, who faced significant challenges accessing benefits [12].

Chile’s experience offers valuable insights for other countries, both within and beyond Latin America, that face similar socioeconomic challenges. For nations with weaker social safety nets, the Chilean case underscores the importance of targeted fiscal stimulus and direct transfers to mitigate economic shocks. Conversely, for high-income nations with more robust welfare systems, Chile highlights the need for long-term policies beyond temporary financial assistance and addressing the structural roots of poverty, particularly in marginalized communities. Moreover, Chile’s comprehensive and high-quality data infrastructure on poverty and mortality provides a rare opportunity to systematically study the socioeconomic impact of the pandemic. This makes Chile not only a model for countries with similar welfare structures but also a valuable case study for nations that lack reliable data to assess poverty dynamics in crisis periods. By leveraging such high-resolution data, this study offers insights that can inform data-driven policy decisions in countries where monitoring poverty changes remains a challenge.

This study analyzes the impact of COVID-19 on income poverty in Chile, with a particular focus on regional disparities, rurality, ethnicity, educational attainment, and immigration. By leveraging data from the Chile National Socioeconomic Characterization Survey (CASEN) for 2017, 2020, and 2022, we assess pre-pandemic, pandemic, and post-pandemic poverty trends and explore spatial clustering of poverty rates using Local Moran’s I statistic. Additionally, logistic regression models are used to identify factors associated with the likelihood of living in poverty hotspots. Our findings provide critical evidence on how Chile’s social policies shaped poverty outcomes during and after the pandemic, highlighting the persistent structural inequalities that must be addressed to achieve long-term poverty reduction.

## Materials and methods

### Data

The Chile National Socioeconomic Characterization Survey (CASEN), administered by the Ministerio de Desarrollo Social (Ministry of Social Development), is essential for formulating social policy in Chile [13]. Established in 1990 and conducted biennially or triennially, CASEN employs a probabilistic, stratified, and multistage sampling method, ensuring a representative cross-section of national, regional, and urban-rural populations. This method captures a broad spectrum of demographic segments, including vulnerable groups such as children, the elderly, immigrants and Indigenous populations. Survey modules comprehensively gather data on critical social determinants, including education, employment, income, health, housing conditions and household and family structure. These are periodically updated to reflect new social policy priorities, such as digital access and environmental concerns. The main objective of the CASEN survey is to estimate poverty and income distribution, as well as to evaluate how the most relevant social and economic programs and policies reach the population.

For this study, we analyzed poverty data at the municipality (comuna) level from the 2017, 2020, and 2022 CASEN surveys (S1 Table). Selecting the years 2017, 2020, and 2022 allows us to capture pre-pandemic, pandemic, and post-pandemic trends, providing a comprehensive overview of how socioeconomic conditions have evolved over this period. Poverty is defined as having an income level below a threshold deemed necessary to achieve a minimum standard of living. The units of analysis for the survey are households and individuals. The household sample sizes for the 2017, 2020, and 2022 surveys were 70,948, 62,911, and 72,056 households, respectively, corresponding to 216,439, 185,437, and 202,231 individuals. The sample includes the calculation of the expansion factor, and all analyses must be conducted using this expansion factor, ensuring that the results are representative of the population residing in private households nationwide. Although the survey is not representative at the municipal level, the SAE (Small Area Estimation) methodology estimates income poverty at the municipality level. The Fay-Herriot (FH) SAE methodology is widely employed to enhance the precision of estimates for small geographic or demographic areas with limited sample sizes. It relies on a model that combines direct survey estimates with administrative data using regression models with random effects [14].

The rich data from CASEN facilitates in-depth analysis of poverty trends and income distribution, thereby serving as a cornerstone for public policy aimed at social development and poverty alleviation. Moreover, it enables policymakers and researchers to track progress, identify vulnerable groups, and allocate resources more efficiently. Furthermore, the information derived from CASEN has become a technical support tool for the design and evaluation of public plans and policies. Adhering to strict ethical standards, CASEN ensures respondent anonymity, with microdata made publicly available to maintain transparency and support independent research. While this study primarily focuses on income-based poverty, alternative approaches such as the Multidimensional Poverty Index (MPI) have been used in other contexts to capture poverty across multiple deprivations, providing a more holistic measure of socioeconomic hardship [15].

### Fiscal stimulus and regional assumptions

Chile implemented a fiscal stimulus program during the pandemic to protect employment and businesses and provide monetary transfers to vulnerable populations. While this program is acknowledged as an important contextual factor, its regional allocation and impact were not explicitly incorporated into the analysis due to the lack of disaggregated data on fiscal transfers at the regional level. Consequently, we do not consider a differential impact of the fiscal stimulus program across regions, which represents a potential limitation. This simplifies the interpretation of regional results but may obscure the heterogeneity in program implementation. Future research could benefit from using Difference-in-Differences (DiD) methods that account for spatial spillovers, as fiscal policies may have indirect effects on neighboring regions that influence local economic recovery [16]. We discuss this limitation further in the discussion section and emphasize the need for future research using granular data on fiscal transfers to examine their regional effects.

### Spatial clustering of poverty rates

We employed a methodology which is widely applicable for analyzing regional disparities in poverty and social inequalities in diverse settings. Specifically, we used the univariate Local Moran’s I statistic to evaluate the trend in spatial clustering of poverty rates in the years 2017, 2020, and 2022 at the municipality level in Chile, utilizing GeoDa [17]. We applied queen’s contiguity weights for the spatial weighting method, considering neighboring municipalities with a common boundary. Subsequently, we identified and displayed significant local indicators of spatial association (LISA) clusters on the maps of Chile, classified as high-high (referred to as Hotspot), high-low, low-high, and low-low (referred to as Coldspot). For example, the high-high (Hotspot) cluster indicates municipalities with high rates of poverty surrounded by other municipalities with high poverty rates. Similarly, the high-low cluster represent municipalities with high poverty rates that are adjacent to municipalities with low poverty rates. Low-high clusters indicate municipalities with low poverty rates adjacent to high poverty rate municipalities. In contrast, low-low (Coldspot) clusters indicate municipalities with low poverty rates surrounded by other low poverty rate municipalities. We used a significance level of 0.05 and a permutation test with 999 permutations to assess the statistical significance of our results. All the spatial analysis and mapping tasks were performed using GeoDa and QGIS software. GeoDa was used to calculate spatial statistics and identify LISA clusters, while QGIS was employed to visualize the spatial distribution of the clusters on detailed maps.

### Multivariable analysis

To identify the magnitude of each factor associated with the risk of being in the Hotspot category and to analyze the differences in these risks, a logistic regression model was implemented for the years 2017 (pre-pandemic) and 2022 (post-pandemic). The dependent variable is coded as 1 if the person lives in a municipality classified as a hotspot and 0 if they belong to the low-low, low-high, or high-low clusters. The independent variables included in the model were all those used in the bivariate analysis: sex, age groups, area of residence, immigration status, belonging to an Indigenous group, and education level (Table 2). While we acknowledge the fiscal stimulus program as a contextual factor, it was not included as a variable in the regression analysis due to the unavailability of detailed data on its regional distribution and effects.

**Table 1:** Cluster characterization of different poverty clusters (High-high (Hotspot), Low-low (Coldspot), Low-high, and High-low) across the years 2017, 2020, and 2022.

Type cluster	N° communes			Population			Poverty (%)		
	2017	2020	2022	2017	2020	2022	2017	2020	2022
High-high (Hotspot)	52	50	56	1,212,785	1,687,947	1,532,379	19.3%	17.0%	13.2%
Low-low (Coldspot)	73	50	69	8,575,738	6,593,427	9,107,151	5.3%	8.0%	4.2%
Low-high	7	1	7	750,713	250593	821,605	9.4%	11.8%	8.4%
High-low	1	6	1	181,785	344793	241,909	14.5%	13.2%	13.5%
**Total included**	**133**	**107**	**133**	**1,072,1021**	**8,876,760**	**11,703,044**	**7.3%**	**10.0%**	**5.9%**
Not statistically significant communes	190	216	201	7,082,069	10665850	8,170,635	10.5%	11.5%	7.4%
Islands without neighbors^*^	1	1	1	4,342	3189	4,894	N/A	N/A	N/A
**Total CASEN**	**324**	**324**	**335**	**17,807,432**	**19,545,799**	**19,878,573**	**8.6%**	**10.8%**	**6.5%**

*Puqueldón island.

**Table 2:** Sociodemographic characteristics of the cluster population in the High-high (Hotspot) and Low-low (Coldspot) clusters for 2017, 2020, and 2022.

Variable	Value	CASEN 2017			CASEN 2020			CASEN 2022			Prevalence ratio 2023/2017	
		Hotspot % (n = 1,212,785)	Coldspot % (n = 8,575,738)	p-value	Hotspot % (n = 1,678,947)	Coldpost % (n = 6,593,427)	p-value	Hotspot % (n = 1,532,379)	Coldpost % (n = 9,107,151)	p-value	Hotspot	Coldspot
**Gender**	Male	47.7	47.6	0.214	45.6	45.9	0.455	49.1	49.4	0.095	1.0	1.0
	Female	52.3	52.4		54.4	54.1		50.9	50.6		1.0	1.0
**Age groups (years)**	0-24	34.5	34.6	<0.001	34.4	33.2	<0.001	32.9	31.6	0.001	1.0	0.9
	25-64	49.5	52.7		50.1	54.2		52.6	56.5		1.1	1.1
	≥ 65	16.0	12.7		15.5	12.6		14.5	11.9		0.9	0.9
**Residence area**	Urban	60.1	96.4	<0.001	73.2	94.6	<0.001	74.5	96.4	<0.001	1.2	1.0
	Rural	39.9	3.6		26.8	5.4		25.5	3.6		0.6	1.0
**Immigrant condition**	Chilean	99.6	93.4	<0.001	98.6%	91.5	<0.001	95.1	87.7	<0.001	1.0	0.9
	Foreigner	0.4	6.6		1.4%	8.5		4.9	12.3		12.3	1.9
**Belonging to native people**	Yes	20.6	6.5	<0.001	22.8	8.2	<0.001	23.2	6.6	<0.001	1.1	1.0
	No	79.4	93.5		77.2	91.8		76.8	93.4		1.0	1.0
Education level (^*^)	Primary (0–8)	36.2	18.3	<0.001	28.1	14.0	<0.001	26.1	13.4	0.001	0.7	0.7
	Secondary (9–12)	42.8	43.8		44.3	39.9		47.1	39.1		1.1	0.9
	13 or more years	21.0	38.0		27.6	46.1		26.1	47.5		1.2	1.3

(

*) Population 15 years or older.

Statistical analyses were conducted using STATA V15 and SPSS V25 software.

## Results

### Cluster analysis

The analysis identified four clusters based on municipality-level poverty for each year considered. These clusters represented 60.2%, 45.4%, and 58.9% of the surveyed population in 2017, 2020, and 2022, respectively, and more than a third of the municipalities included in the analysis (41% in 2017 and 2022, and 33% in 2020). The Low-high and High-low clusters included a small number of municipalities each year, with the majority concentrated in the High-high (Hotspot) and Low-low (Coldspot) clusters, which are the primary focus of our analysis (Table 1).

The High-high (Hotspot) cluster maintained around 50 municipalities in all three surveys, with the proportion of its population rising from 6.8% in 2017 to 8.6% in 2020 and then decreasing to 7.7% in 2022. The poverty rate within the Hotspot was 19.3% in 2017, 17% in 2020, and 13.2% in 2022. In contrast, the Low-low (Coldspot) cluster included more municipalities than the Hotspot cluster in 2017 and 2022, representing over 40% of the population in those years (48.2% in 2017, 33.7% in 2020, and 45.8% in 2022), with poverty rates consistently below national figures (5.3%, 8%, and 4.2% in 2017, 2020, and 2022, respectively).

### Spatial Distribution of Clusters

**Fig 1:**
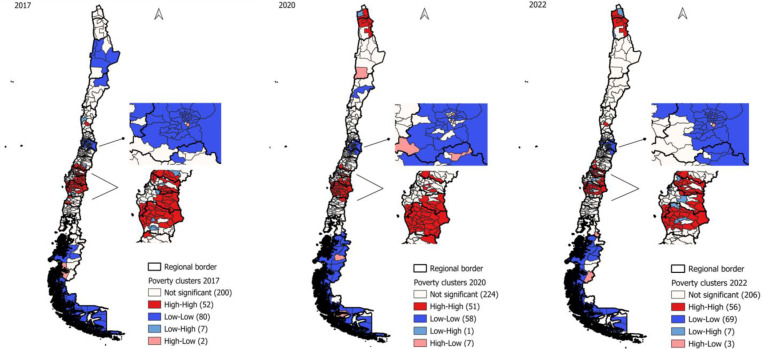
Spatial Distribution of Poverty Clusters in Chile (2017, 2020, 2022). The geographical distribution of poverty clusters in Chile over three years: 2017, 2020, and 2022. Our analysis focuses on High-high (Hotspot) and Low-low (Coldspot) clusters based on the concentration of poverty within the municipalities.

The clusters’ spatial distribution and variation before, during, and after the COVID-19 pandemic revealed significant regional disparities. As shown in Fig 1, in 2017, Coldspot clusters were identified in the northern region (known for large-scale copper mining), the central region (including the national capital and main ports), and the southernmost part of Chile (a region with high tourism potential and oil production). The Hotspot clusters in 2017 were predominantly in the central-southern regions, where a high concentration of the Mapuche ethnic group resides, and poverty rates have persistently been high over time.

These consistent geographic patterns in poverty continued during and after the pandemic. However, starting in 2020, a new Hotspot cluster emerged in the northernmost part of the country, an important immigration hub and entry point into Chile, both legally and illegally.

Conversely, from 2020 onwards, the Coldspot cluster disappeared from the northern region (copper mining area), remaining only in the capital and southern regions.

### Temporal variation of cluster characteristics

The analysis of the sociodemographic characteristics of these clusters revealed some differences and highlighted social determinants (Table 2). There were no significant sex differences in both clusters throughout the period. All other characteristics showed statistically significant differences.

In the Hotspot clusters, the proportion of people aged over 65 was higher than in Coldspot clusters (16% vs. 12.7% in 2017, 15.5% vs. 12.6% in 2020, and 14.5% vs. 11.9% in 2022), while the proportion of working-age adults (25–64 years) was lower (49.5% vs. 52.7% in 2017, 50.1% vs. 54.2% in 2020, and 52.6% vs. 56.5% in 2022).

A higher share of the Hotspot cluster lives in rural areas (39.9% vs. 3.6% in 2017, 26.8% vs. 5.4% in 2020, and 25.5% vs. 3.6% in 2022). However, comparing prevalences from 2017 to 2022, there was a decrease in rurality within Hotspot clusters while it remained stable in Coldspot clusters.

The Hotspot clusters had a consistently lower share of immigrants than the national average (0.4% vs. 6.6% in 2017, 1.4% vs. 8.5% in 2020, and 4.9% vs. 12.3% in 2022). Nevertheless, the increase in the prevalence of the foreign population between 2017 and 2022 was significantly higher in Hotspot clusters compared to Coldspot clusters.

Hotspot clusters had a higher proportion of Indigenous people than the national average across all three survey years (20.6% vs. 6.5% in 2017, 22.8% vs. 8.2% in 2020, and 23.2% vs. 6.6% in 2022). The share of the population having only primary education were higher in Hotspot clusters than in the national average (36.2% vs. 18.3% in 2017, 28.1% vs. 14% in 2020, and 26.1% vs. 13.4% in 2022). Conversely, the percentages of individuals with higher education were lower in Hotspot clusters than in the nation as a whole (21% vs. 38% in 2017, 27.6% vs. 46.1% in 2020, and 26.1% vs. 47.5% in 2022). Between 2017 and 2022, there was a decrease in the prevalence of primary education (prevalence ratio = 0.7 in both clusters) and an increase in higher education (prevalence ratios of 1.2 in Hotspot and 1.3 in Coldspot clusters).

### Multivariable analysis

**Fig 2:**
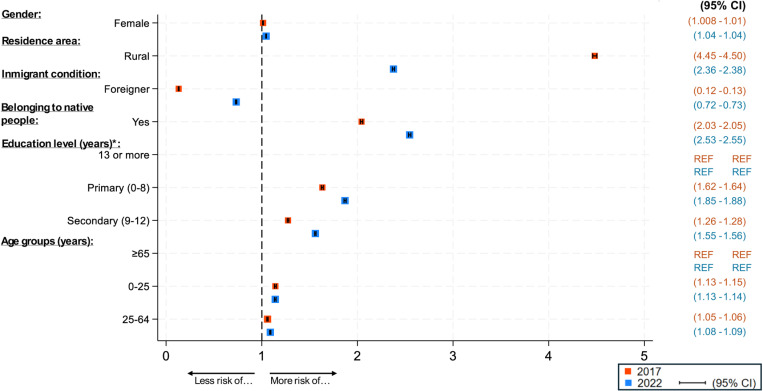
Adjusted Odds Ratios for Determinants of Hotspot Cluster Membership (2017 and 2022). Adjusted odds ratios (OR) for various sociodemographic determinants influencing the likelihood of a municipality being classified as part of a High-high (Hotspot) cluster in 2017 and 2022.

Two multivariable models were developed, one for 2017 and the other for 2022, to jointly analyze the sociodemographic determinants and obtain adjusted odds ratios (OR) for the likelihood of belonging to the Hotspot cluster each year. While the share of immigrants in a municipality significantly reduced the likelihood of belonging to the Hotspot cluster in both years, all other variables, significantly increased the likelihood of belonging to the Hotspot cluster (Fig 2). The most influential variables were rurality and Indigenous status. However, while the influence of rurality decreased (OR = 4.48 in 2017 and OR = 2.37 in 2022), the influence of Indigenous status increased (OR = 2.04 in 2017 and OR = 2.54 in 2022), becoming the most significant factor in 2022. The protective factor of the share of immigrants was lower in 2022 than in 2017 (OR = 0.13 in 2017 and OR = 0.73 in 2022). This is consistent with the increased prevalence of the foreign population in the hotspot between 2017 and 2022 (give these shares here).

## Discussion

The key findings of this study highlight the complex impact of the COVID-19 pandemic on poverty in Chile. The analysis revealed that while Chile experienced a significant reduction in income poverty from 2020 to 2022 due to various monetary subsidies and social assistance programs, regional disparities persisted. Chile’s social insurance system, one of the most developed in Latin America, played a crucial role in mitigating poverty through emergency cash transfers (Ingreso Familiar de Emergencia, IFE), pension withdrawals, and wage subsidies. These interventions helped reduce poverty from 10.8% in 2020 to 6.5% in 2022. However, their effectiveness was not uniform across all regions and demographic groups, with similar concerns raised in Argentina, where the pension system experienced substantial strain during the pandemic [18]. Rural areas, regions with high labor informality (e.g., Araucanía and northern border zones), Indigenous populations, and migrant communities faced disproportionate economic hardship due to barriers in accessing formal social protections. Our spatial analysis highlights persistent poverty hotspots in these areas, suggesting that while national-level trends show poverty reduction, localized vulnerabilities remain.

Despite Chile’s well-developed social safety net—the strongest in Latin America by many measures [1]—the pandemic highlighted its limitations in fully mitigating the economic impacts for marginalized populations. Indigenous communities, rural residents, and informal workers, who already faced systemic disadvantages, were disproportionately affected, reflecting broader trends observed in other Latin American countries [2]. Unlike high-income countries with comprehensive wage subsidies and reinforced social security programs (e.g., Germany and Scandinavian nations), Chile’s response relied more on temporary financial assistance than long-term structural social protections. The widespread use of pension withdrawals, while providing immediate economic relief, may have long-term consequences for financial security, particularly for lower-income populations. In contrast, European countries prioritized employment protection schemes, ensuring greater income stability during the crisis. This distinction underscores the limitations of Chile’s social insurance framework, which provided short-term poverty alleviation but did not sufficiently address structural inequalities.

The results of this study allow for the analysis of the impact of the COVID-19 pandemic on the magnitude of income poverty in Chile. Although the country had been experiencing a sustained decrease in poverty rates [11], 2020 saw an increase of nearly 10% due to gross domestic product (GDP) contraction and massive job losses [19]. This pattern was also observed throughout Latin America and the Caribbean, which was identified as the most affected developing region by the pandemic [20]. The World Bank’s 2022 report on poverty and shared prosperity emphasizes that such disruptions disproportionately impact the most vulnerable populations and highlights the importance of correcting course through targeted policy interventions that promote sustainable economic growth and social equity [21].

The unemployment rates in Chile for the years 2017, 2020, and 2022 highlight the economic fluctuations and impacts of the COVID-19 pandemic. In 2017, the unemployment rate was 6.7%, reflecting a relatively stable economic period. By 2020, due to the economic disruptions caused by the COVID-19 pandemic, including widespread job losses and business closures, the unemployment rate increased by 4 percentage points to 10.6%. By 2022, as the economy began to stabilize and recover from the pandemic’s impacts, the unemployment rate fell to 7.8% but did not reach the pre-pandemic level at 6.7% [22]. The lessons from Chile’s response, including implementing fiscal stimulus programs and monetary transfers [23–25], provide valuable insights for other countries with less developed social insurance systems. However, Chile’s high-quality socioeconomic data provides a unique opportunity to analyze these poverty dynamics in greater detail. Many countries in the region lack the granular data necessary to assess the localized effects of the pandemic on poverty, making Chile a valuable case study for understanding how targeted fiscal policies interact with structural inequalities.

Nevertheless, our study primarily examines disparities by region and their demographics, with our findings pointing to broader implications for designing future policies. Given the uneven impact of Chile’s social insurance measures, future interventions should focus on improving accessibility for rural, Indigenous, and migrant populations by expanding formal protections beyond emergency cash transfers. Targeted investments in education, healthcare, and employment opportunities are critical for reducing intergenerational poverty, particularly in rural and Indigenous communities. Additionally, integrating informal workers into the formal economy through policy incentives and labor protections would enhance economic resilience in future crises.

Additionally, the results from this study are relevant to countries with more developed social insurance systems. While such systems may offer better short-term protection against income shocks, the persistence of structural inequalities, such as those related to ethnicity and rurality, can undermine the long-term effectiveness of social protections. For instance, the disproportionate burden borne by Indigenous populations and rural communities in Chile demonstrates that even advanced social systems must adopt a multidimensional approach to tackle these challenges. Policymakers in other settings can draw from these findings to design interventions that complement existing safety nets by addressing structural inequalities at their root. These results highlight that equitability and fairness—while political choices—must be operationalized through evidence-based policies that target systemic barriers to progress.

Our study’s results also allow for a deeper examination of more structural aspects of poverty, identifying factors in the three analyzed periods, such as rurality, ethnicity, and immigration. The relationship between rurality and poverty is not new; CEPAL recognizes the higher incidence of poverty in rural areas as a structural characteristic of Latin America and the Caribbean due to lower wages and higher informality [20]. The impact of the pandemic on the evolution of various forms of rural poverty has also been noted [26]. In Chile, the reduction in rural-urban poverty disparities observed during the pandemic suggests that temporary measures helped mitigate these inequalities. However, as seen in other countries, structural challenges related to rural livelihoods, including limited access to education and healthcare, require long-term strategies for sustainable progress [11].

A similar situation occurs with Indigenous peoples. The share of the population identifying with an Indigenous ethnicity was higher in the Hotspot cluster and consistently showed higher poverty rates. However, since 2020, a reduction in the gap has been observed (reaching 8.8% vs. 6.2% for Indigenous and non-Indigenous groups in 2022) [25]. This temporary reduction in disparities is likely attributable to pandemic-related support programs targeting the most vulnerable populations. However, as evidenced by studies from other countries, such measures are insufficient to address the systemic disadvantages faced by Indigenous communities. The Chilean case highlights the global importance of designing policies prioritizing long-term structural change for Indigenous populations [11].

A third determinant, emerging more strongly since 2020, is immigration. While the proportion of immigrants was higher in the Coldspot cluster than in the Hotspot, the significant increase observed in the latter must be analyzed considering recent migration patterns, marked by intraregional migration and crises in Haiti and Venezuela [27,28], which led to an increase in the migrant population, a change in the countries of origin, and a worsening of the poverty gap. Before 2017, the immigrant population was characterized by lower poverty levels than the Chilean population; since then, the situation has reversed [29]. Additionally, an unequal socioeconomic impact of the pandemic has been observed, worsening conditions for Venezuelan, Haitian, and Peruvian immigrants [30]. In 2022, the income poverty rate for foreign-born individuals nearly doubled that of native-born Chileans (11% vs. 6%) [31]. The Chilean experience demonstrates the need for inclusive social policies that address the specific vulnerabilities of immigrant populations, particularly during times of crisis, a lesson applicable to other nations managing large-scale migration flows.

Poverty hotspots are distinguished as areas where poor municipalities are surrounded by other poor municipalities, creating a cycle of vulnerability and social marginalization [32]. One of these hotspots is located in the central-southern region of the country, while a second emerged in the far north starting in 2020. Given the previously discussed characteristics of poverty, it is unsurprising that both hotspots are located in areas with a significant presence of Indigenous peoples, predominantly the Aymara in the north and the Mapuche in the central south [13,33]. The central-southern hotspot is particularly characterized by high rurality, compounded by a dense Indigenous presence, geographic isolation, and limited access to social services. This setting exacerbates social exclusion and economic hardship. Moreover, the ongoing conflict between the Chilean state and the Mapuche people over Indigenous land rights continues to deepen these disparities, further entrenching economic and social inequalities in the region [34,35].

In the north, the presence of Indigenous peoples, historically linked to migration from neighboring countries, has been compounded in recent years by the settlement of immigrants from more distant places such as Colombia and Venezuela. Their often illegal entry has also led to significant conflicts, even requiring a military presence in the area [36]. This recent migration, associated with higher poverty and social insecurity, may explain the emergence of part of the Hotspot cluster from 2020 and the disappearance of the Coldspot cluster in the area [27,29].

We acknowledge a limitation in our analysis regarding the implementation of Chile’s fiscal stimulus program, designed to protect employment and businesses and provide monetary transfers to vulnerable populations. Specifically, the analysis assumes homogeneity in the impact of monetary transfers across regions, as disaggregated data on these transfers’ precise allocation and regional effects were unavailable. This assumption may have overlooked potential heterogeneities in program implementation and its influence on observed poverty outcomes. Future research should aim to explore the differential impacts of fiscal policies at a more granular level, incorporating regional data where available. Such analyses could clarify how fiscal stimulus programs interact with structural inequalities to influence poverty outcomes during crises. Furthermore, the binary poverty indicator has limitations, as it fails to capture the social gradient of deprivation. The Multidimensional Poverty Index (MPI) assesses poverty across education, health, and living standards, offering a broader perspective but requiring extensive non-income data often unavailable at the municipal level [37–39]. Despite its constraints, income-based poverty measurement remains widely used for comparability across regions and policy alignment [40,41]. In Chile, the SAE methodology enhances municipal-level estimates, ensuring spatial precision.

Household surveys have several strengths. Firstly, they allow for the collection of detailed data at the individual and family levels, providing a comprehensive view of the socioeconomic conditions of the studied populations [42]. Additionally, the 2–3 year periodicity of these surveys, like CASEN in Chile, facilitates tracking changes over time, allowing for trend analysis and evaluation of public policy impacts [43]. Another key strength is their ability to capture various variables, enabling multidimensional and in-depth analyses of household welfare and income [44]. However, there are also weaknesses, the main one being self-report bias, where data can be inaccurate [45]. In addressing gross and net response rates and potential non-response biases, it is essential to note that the household surveys utilized in this study, like the CASEN survey in Chile, typically achieve high response rates, ensuring robust data quality. The CASEN survey, for instance, is known for its comprehensive approach and efforts to minimize non-response bias through rigorous follow-up protocols and the use of weighting adjustments to account for any remaining biases. Despite these measures, potential non-response bias can still exist, particularly among hard-to-reach populations such as those in remote rural areas or among recent immigrants. To ensure the representativeness of the samples, the survey employs stratified sampling methods to match the population’s key characteristics, such as age, sex, ethnicity, and geographic location. This approach allows for a representative snapshot of the population, facilitating accurate analysis of socioeconomic conditions.

Overall, our results highlight the need for a multidimensional approach to poverty alleviation that goes beyond immediate financial assistance. Long-term solutions must focus on enhancing education, healthcare, employment opportunities, and social security, particularly for the most vulnerable populations such as rural residents, Indigenous peoples, and immigrants. Policies should aim to promote inclusive economic growth and strengthen social protections to build a more resilient society capable of handling future crises. Our work also underscores the importance of secondary data sources from representative household surveys of the country’s population that collect information on socioeconomic status, living conditions, and access to various public plans and policies. This data is crucial for monitoring the evolution of social and economic situations and living conditions in countries, especially those in developing stages.

Chile’s experience highlights the need to expand formal social protections to better cover informal workers and migrants, ensuring equitable access to economic support during crises. Strengthening rural development policies—through investments in infrastructure, education, and employment opportunities—is crucial to addressing persistent poverty hotspots. Compared to high-income nations that implemented robust labor protections and automatic stabilizers, Chile’s pandemic response was more reactive, relying on temporary financial aid rather than long-term structural interventions. Moving forward, policymakers should prioritize comprehensive social insurance reforms that enhance economic resilience, reduce labor market informality, and integrate data-driven decision-making to better target vulnerable populations.

In conclusion, our analysis underscores the importance of integrating these insights into actionable, long-term policy frameworks. Addressing poverty requires shifting from short-term financial aid to systemic reforms prioritizing equity and resilience. This includes improving data collection to better understand regional and demographic disparities, fostering inclusive economic growth, and ensuring that public policies explicitly address the root causes of inequity. Such steps are essential not only for Chile but for other nations grappling with similar challenges.

## Supporting information

S1 Table Municipality (Comuna) level Poverty (2017, 2020, 2022) obtained from the Chile National Socioeconomic Characterization Survey (CASEN). This table lists the comuna-level poverty estimates used in our analysis.
(XLSX)

## References

[1] Long A, Ascent D. World economic outlook. International Monetary Fund. 2020;177.

[2] Cepal N. Preliminary Overview of the Economies of Latin America and the Caribbean 2020. ECLAC; 2021.

[3] UN. Poverty in Latin America Returned to Pre-Pandemic Levels in 2022, ECLAC Reports with an Urgent Call for Progress on Labour Inclusion 2023. Available from: https://www.cepal.org/en/pressreleases/poverty-latin-america-returned-pre-pandemic-levels-2022-eclac-reports-urgent-call

[4] OECD. OECD Economic Surveys: Chile 2018. 2018.

[5] United Nations Department of Economic and Social Affairs. World Social Report 2021: Reconsidering Rural Development. 2021.

[6] Lustig N, Pabon VM, Sanz F, Younger SD. The impact of COVID-19 lockdowns and expanded social assistance on inequality, poverty and mobility in Argentina, Brazil, Colombia and Mexico. Covid Economics, Vetted and Real-Time Papers. 2020;46:32–67.

[7] Segatto CI, Santos dos FBP, Bichir RM, Morandi EL. Inequalities and the COVID-19 pandemic in Brazil: analyzing un-coordinated responses in social assistance and education. Policy and Society. 2022;41(2):306–20. doi: 10.1093/polsoc/puac005

[8] Poy S, Robles R. Informality, social protection and welfare during the COVID-19 crisis in four Latin American countries. CEPAL Review. 2023;2023(140):21–39. doi: 10.18356/16840348-2023-140-2

[9] World Bank. Chile Overview: Development news, research, data 2021. Available from: https://www.worldbank.org/en/country/chile/overview

[10] Ramos FM, Lara J. COVID-19 and poverty vulnerability. 2022.

[11] Ministerio de Desarrollo Social y Familia. Presentación de Resultados: Encuesta de Caracterización Socioeconómica Nacional (CASEN) 2022. Observatorio Social. 2023. Available from: https://observatorio.ministeriodesarrollosocial.gob.cl/storage/docs/casen/2022/Presentaci%C3%B3n_Resultados_Casen_2022%20_v20oct23.pdf

[12] Busso M, Camacho J, Messina J, Montenegro G. Social protection and informality in Latin America during the COVID-19 pandemic. PLoS One. 2021;16(11):e0259050. doi: 10.1371/journal.pone.0259050 34735496 PMC8568185

[13] Ministry of Social Development and Family. Encuesta Nacional de Caracterización Socioeconómica Nacional (CASEN) 2022. Available from: https://observatorio.ministeriodesarrollosocial.gob.cl/encuesta-casen

[14] Porter A.T., Holan S.H., Wikle C.K., Cressie N. Spatial Fay–Herriot models for small area estimation with functional covariates. Spat Stat. 2014;10:27–42.

[15] Alkire S, Santos ME. A Multidimensional Approach: Poverty Measurement & Beyond. Soc Indic Res. 2013;112(2):239–57. doi: 10.1007/s11205-013-0257-3

[16] Butts K. Difference-in-differences estimation with spatial spillovers. arXiv preprint arXiv:210503737. 2021.

[17] Anselin L. Local Indicators of Spatial Association—LISA. Geographical Analysis. 1995;27(2):93–115. doi: 10.1111/j.1538-4632.1995.tb00338.x

[18] Rofman R, Baliña J, López Méndez E. Evaluating the impact of COVID-19 on pension systems in Latin America and the Caribbean: The case of Argentina. IDB Working Paper Series; 2022.

[19] Ministerio de Desarrollo Social y Familia. Análisis de Carencias de la Pobreza Multidimensional en Pandemia 2021. Available from: https://observatorio.ministeriodesarrollosocial.gob.cl/storage/docs/casen/2020/210707_Carencias_PM_Casen_en_Pandemia_2020.pdf

[20] CEPAL. Panorama Social de América Latina. 2020.

[21] World Bank. Poverty and Shared Prosperity 2022.

[22] World Bank Group. Unemployment, total (% of total labor force) (modeled ILO estimate) - Chile. Available from: https://data.worldbank.org/indicator/SL.UEM.TOTL.ZS?locations=CL

[23] Programa de las Naciones Unidas para el Desarrollo en Chile. Análisis de la Pobreza por ingresos en Chile post-pandemia: Logros y Desafíos Pendientes 2023. Available from: https://www.undp.org/sites/g/files/zskgke326/files/2023-08/analisis_pobreza_2023_pnud_chile.pdf

[24] Ministerio de Hacienda. Avances en Políticas Económicas y Sociales. 2021. Available from: https://biblio.hacienda.cl/avances-en-politicas-economicas-y-sociales-2021

[25] Ministerio de Desarrollo Social y Familia. Resultados de Pobreza por Ingresos. Encuesta de Caracterización Socioeconómica Nacional 2022. Available from: https://observatorio.ministeriodesarrollosocial.gob.cl/storage/docs/casen/2022/Resultados%20pobreza%20por%20ingresos%20Casen%202022.pdf

[26] Clausen J. Pobreza rural en América Latina y el Caribe en el contexto del COVID-19-Cinco líneas de incidencia y acción para no dejar a ningún territorio rural atrás. Santiago de Chile; 2022. Contract No.: nº 36.

[27] Colmenares N, Abarca K. La migración a nivel local en Chile. Desafíos, demandas y políticas en tiempos de pandemia. Si Somos Am. 2022;22(1). doi: 10.4067/s0719-09482022000100164

[28] Freier LF, Parent N. A South American Migration Crisis: Venezuelan outflows test neighbors’ hospitality. Migration Policy Institute; July 18 2018.

[29] Banco Internacional de Reconstrucción y Fomento/Banco Mundial. Superación de la pobreza y acceso a la vivienda: desafíos estructurales a abordar para la integración de la población migrante y refugiada en Chile. 2023. Available from: https://documentos.bancomundial.org/es/publication/documents-reports/documentdetail/099121923182524486/P17578012041b70b1193261ce08b4e77106

[30] Soto-Alvarado S, Morollón JP, Gil-Alonso F. Impacto diferencial de la COVID-19 sobre la pobreza e ingresos de inmigrantes y nativos: el caso de Chile. REMHU: Revista Interdisciplinar da Mobilidade Humana. 2024;32;e321890.

[31] Ministerio de Desarrollo Social y Familia. Pobreza y migración Desde la Casen 2022. Available from: https://serviciomigraciones.cl/wp-content/uploads/2023/12/Analisis-Casen-pobreza-migracion-SERMIG.pdf

[32] Pereira P. Spatial Segregation: The Persistent and Structural Features of Exclusionary Policies. In Leal Filho W, Marisa Azul A, Brandli L, Lange Salvia A, Özuyar PG, Wall T, Editors. Peace, Justice and Strong Institutions. Encyclopedia of the UN Sustainable Development Goals. Springer; 2021.

[33] Instituto Nacional de Estadísticas. Sínteisis de Resultados. Censo 2017. Available from: https://www.ine.gob.cl/docs/default-source/censo-de-poblacion-y-vivienda/publicaciones-y-anuarios/2017/publicaci%C3%B3n-de-resultados/sintesis-de-resultados-censo2017.pdf?sfvrsn=1b2dfb06_6

[34] Rodríguez-Garcés CR, Padilla-Fuentes G, Suazo-Ruíz C. Etnia mapuche y vulnerabilidad: una mirada desde los indicadores de carencialidad socioeducativa. Encuen. 2020;18(01). doi: 10.15665/encuent.v18i01.2232

[35] Andrade MJ. La lucha por el territorio mapuche en Chile: una cuestión de pobreza y medio ambiente. L’Ordinaire des Amériques. 2019(225).

[36] Ministerio de Relaciones Exteriores. Situación migratoria en la Macro Zona Norte de Chile February 2022. Available from: https://www.minrel.gob.cl/noticias-anteriores/situacion-migratoria-en-la-macro-zona-norte-de-chile

[37] Alkire S, Foster J. Counting and multidimensional poverty measurement. Journal of Public Economics. 2011;95(7–8):476–87. doi: 10.1016/j.jpubeco.2010.11.006

[38] Santos ME, Villatoro P. A Multidimensional Poverty Index for Latin America. Review of Income and Wealth. 2016;64(1):52–82. doi: 10.1111/roiw.12275

[39] United Nations Development Programme. Global multidimensional poverty index, 2022: unpacking deprivation bundles to reduce multidimensional poverty. United Nations Development Programme and Oxford Poverty and Human Development Initiative; 2022.

[40] Ferreira FH, Messina J, Rigolini J, López-Calva L-F, Lugo MA, Vakis R, et al. Economic mobility and the rise of the Latin American middle class. World Bank Publications; 2012.

[41] Ravallion M. The economics of poverty: History, measurement, and policy: Oxford University Press; 2015.

[42] Glewwe P, Grosh ME. Designing household survey questionnaires for developing countries: lessons from 15 years of the living standards measurement study. World Bank; 2000.

[43] Instituto Nacional de Estadísticas (INE). Encuesta de Presupuestos Familiares (EPF) 2021-2022. Chile; 2022.

[44] World Bank. Poverty Reduction in Latin America: An Assessment. World Bank Report. 2023.

[45] Bound J, Brown C, Mathiowetz N. Measurement error in survey data. Handbook of econometrics. 5: Elsevier; 2001. p. 3705–843.

